# Comparing sagittal plane kinematics and kinetics of gait and stair climbing between hypermobile and non-hypermobile people; a cross-sectional study

**DOI:** 10.1186/s12891-021-04549-2

**Published:** 2021-08-19

**Authors:** Alexander Vernon Bates, Alison H. McGregor, Caroline M. Alexander

**Affiliations:** 1 Department of Surgery and Cancer, Imperial College London, London, UK; 2 Department of Therapies, Imperial College Healthcare NHS Trust, Charing Cross Hospital, London, W6 8RF UK; 3 Department of Physiotherapy, Imperial College Healthcare NHS Trust, London, UK

## Abstract

**Background:**

Joint Hypermobility Syndrome (JHS) presents with a range of symptoms including widespread joint hypermobility and chronic arthralgia. The study objective was to investigate whether impairments in JHS are due to hypermobility or another factor of JHS by identifying impairments in gait and stair-climbing tasks; an activity that is demanding and so may better show differences between the cohorts.

**Methods:**

Sixty-eight adults participated; 23 JHS, 23 Generalised Joint Hypermobility (GJH), and 22 Normal Flexibility (NF). Inclusion criteria for JHS participants were a positive classification using the Brighton Criteria, for GJH a Beighton Score ≥ 4, and for NF a Beighton Score < 4 with no hypermobile knees. Participants were recorded with a 10-camera Vicon system whilst they performed gait and stair-climbing. Temporal-spatial, and sagittal plane kinematic and kinetic outcome measures were calculated and input to statistical analyses by statistical parametric mapping (SPM).

**Results:**

During the gait activity JHS had significantly greater stride time and significantly lower velocity than NF, and significantly greater stride time, lower velocity, and lower stride length than GJH. SPM analysis showed no significant differences between groups in gait kinematics. There were significant differences between groups for gait moments and powers; people with JHS tended to have lower moments and generate less power at the ankle, and favour power generation at the knee. A similar strategy was present in stair ascent. During stair descent people with JHS showed significantly more hip flexion than people with NF.

**Conclusions:**

As there was only one significant difference between GJH and NF we conclude that impairments cannot be attributed to hypermobility alone, but rather other factor(s) of JHS. The results show that both gait and stair-climbing is impaired in JHS. Stair-climbing results indicate that JHS are using a knee-strategy and avoiding use of the ankle, which may be a factor for clinicians to consider during treatment.

**Supplementary Information:**

The online version contains supplementary material available at 10.1186/s12891-021-04549-2.

## Background

Symptomatic hypermobility, here called Joint Hypermobility Syndrome (JHS), is a Heritable Disorder of Connective Tissue (HDCT) characterised by multiple hypermobile joints associated with chronic pain [1]. Whilst the main symptom is long-term and widespread pain [1], JHS is a multi-factorial condition that presents with a wide range of articular and extra-articular symptoms, including joint instability such as recurring and traumatic joint dislocations, and proprioceptive differences [2, 3]. Although JHS is rare (prevalence estimated to 0.75–2% in the general population [2]), having multiple hypermobile joints with little to no associated symptoms is common (approximately one in five women in the UK [4]) and has been defined as Generalised Joint Hypermobility (GJH). Historically one way of classifying JHS is the Brighton Criteria; patients are classified as JHS if they do not have any other HDCT and meet a set number of major and minor criteria [5]. Part of the Brighton Criteria is the Beighton Score which is used to classify GJH^1^. In the Beighton Score nine joints are tested for hypermobility and if four or more are hypermobile then the person is classified as GJH. Classification of JHS has historically been a challenge [6]. Recently a new classification system was proposed where the most severely symptomatic individuals are classified as Hypermobile Ehlers Danlos Syndrome (hEDS) and patients exhibiting fewer and/or less severe symptoms classified as Hypermobility Spectrum Disorder (HSD) [7, 8]. In this paper we use the legacy terms of JHS and GJH as this research was conducted prior to the proposal of the new classification system.

Although JHS is rare in the general population the prevalence within healthcare clinics is high; 39% in a UK pain clinic [6] and 45% of patients at a West-London general rheumatology clinic [9]. The high proportion of patients presenting in healthcare with JHS combined with repeated visits to healthcare means this is an important cohort to treat. A mainstay of treatment for the physical symptoms is physiotherapy [10]. Unfortunately physiotherapy is not always effective, with uncertainty about what type of physiotherapy should be prescribed, a lack of awareness by some healthcare practitioners [10], and patients not always rating physiotherapy as improving symptoms [11]. One part of the problem is the uncertainty about what causes the symptoms (specifically pain) in the JHS cohort. Researchers have suggested that improving strength, instability, proprioception, and correcting badly adapted posture/movements will improve symptoms of JHS [12]. However, identifying and targeting these suspected movement dysfunctions during functional tasks is challenging. Therefore, identifying potential functional impairments may give clinicians insight into where to target treatment.

There have been several studies of movement in hypermobile cohorts which we summarised in a previous systematic review [13]. We found that most studies investigated gait, however, this was often reported differently with a wide variety of outcome measures. A conclusion of the review was there were few common findings between studies, which could be due to vagaries in reporting of the power of the studies and how sample sizes were determined [13]. This was recently addressed by a comprehensive study of lower-limb kinematics and kinetics in JHS by Alsiri et al. [14] who compared JHS to a control group without hypermobile joints and found that JHS participants had significant reductions in temporal-spatial measures of gait; walking speed, stride length, and step length. They also found JHS had significantly reduced maximum knee flexion during swing phase, and some reduced joint moments and power generation. They propose these differences indicate a ‘stiffening’ walking pattern to avoid pain and improve balance. However, due to the wide range of symptoms associated with JHS it is difficult to tease out what is causing impairments. It could be pain, which has been shown to alter walking kinematics, the hypermobility itself, muscle weakness, reduced proprioception, or fear of falling, or a combination of the many factors inherent in JHS. Although there are several studies of gait there are relatively few other studies investigating other activities of daily living.

Importantly, all these studies compared JHS groups to normal flexibility (NF) control groups. Due to the multi-factorial nature of JHS these studies could not conclude whether it was the hypermobility per se causing the observed impairments, or whether it was another factor of the syndrome. This study builds on the current knowledge in two ways. Firstly, we use an additional GJH control group (i.e. a group with similar flexibility but no symptoms). By comparing JHS to both GJH and NF, we aim to discover whether functional impairments are unique to JHS or are caused by the underlying hypermobility. Secondly, we include a stair-climbing activity. We chose stair-climbing for two reasons. Firstly, both stair-ascent and stair-descent place greater demands on the body than level walking [15–17]. For example, during stair-ascent, knee joint moments have been found to be up to 25% greater than level walking [16]. Therefore, stair climbing could potentially better highlight impairments in people with JHS where healthcare practitioners could direct treatment. This is particularly relevant for people with JHS as they are already understood to be weak, therefore a more demanding task may reveal greater insights. Secondly, stair-climbing features in one of the questions in the Bristol Impact of Hypermobility questionnaire (BIoH), a questionnaire designed to measure the impact JHS has on a patient [18]. Part of the design process of the BIoH involved asking JHS patients what activities they found challenging. Since stair-climbing features in the final BIoH questionnaire, it is reasonable to say that people with JHS find it a challenging activity. Therefore, the aim of this study was to identify impairments and differences in movement in people with JHS during gait and stair-climbing activities by comparing a JHS group to both GJH and NF groups. This information will indicate whether it is hypermobility or another factor of JHS that causes impairments in movement and will enable clinicians to target treatment appropriately.

## Methods

### Participants

Ethical approval was granted by National Research Ethics Service London-West Ethics Committee. Inclusion criteria were listed in a previous manuscript [19]. Inclusion criteria for all groups were people aged 18–55 who were able to walk unshod and unaided for more than 6 m, no history of lower limb surgery, no present injury, and no neurological or medical condition not associated with JHS. Inclusion criteria for people with JHS was a Beighton Score ≥ 4 and a positive classification of JHS using the Brighton Criteria [2]. Inclusion criteria for people with GJH group were a Beighton Score ≥ 4, and a negative classification for JHS using Brighton Criteria. In addition, bothe people with JHS and people with GJH were required to score positively for at least one knee being hypermobile to participate. Inclusion criteria for people with NF was a Beighton Score < 4, a negative classification for JHS using Brighton Criteria, and no hypermobility in either knee. People with GJH and people with NF were excluded if they reported any lower limb pain. Sample size was informed by the variability of kinematics of hypermobile movement reported in a previous reliability study [20], indicating a minimum sample size of 20 participants in each group were required to reach sufficient power where α = 0.05 (β = 0.2). JHS participants were recruited from Ehlers-Danlos Support UK, The Hypermobile Syndromes Association, and patients from a London NHS Hospital Group. GJH and NF participants were recruited from posters displayed in the hospital and local area.

Twenty-three people with JHS, 23 people with GJH, and 22 NF people were recruited. There were no adverse events during the study. Table 1 lists participant characteristics.

**Table 1:** Participant characteristics of each group (mean ± standard deviation)

	JHS (*n* = 23)	GJH (*n* = 23)	NF (*n* = 22)
Age (years)	33 ± 9	28 ± 6	28 ± 5
Sex (f/m)	20/3	19/4	16/6
Height (cm)	169 ± 8	169 ± 10	172 ± 8
BMI	25.5 ± 5.6	22.9 ± 4.4	22.0 ± 2.8
Beighton Score	6.8 ± 2.1	6.6 ± 1.3	0.3 ± 0.7

### Task and procedure

Testing took place at the Imperial College movement analysis laboratory at Charing Cross Hospital. We affixed reflective markers to participants lower limbs and pelvis, joint angles were calculated using a previously defined model which its reliability has specifically been tested with a hypermobile cohort [20]. Participants were recorded using a 10-camera Vicon system sampling at 100 Hz. Participants performed all tasks barefoot.

For the gait task we asked participants to walk at a self-selected pace along a 6 m walkway which had two force plates embedded (Kistler Instruments Corp., Amherst, USA) sampling at 1000 Hz. Five strides from foot strike to ipsilateral foot strike were used in analysis to determine mean parameters over the gait cycle. Only steps where the foot made full contact with the force plate were used.

For the stair task we asked participants to climb and descend an instrumented staircase comprising of three steps. Step height was 25 cm, width 60 cm and tread depth 25 cm. At the top of the stair was a platform measuring 60 by 60 cm. The Kistler force plates were inserted into the first and second step. Stair-climbing is challenging to explore due to the variable nature of the ways people achieve the task. However, as it is a task identified as problematic for people with JHS it was important to overcome these issues by defining the way the task is achieved. To ensure data was comparable, we asked participants to climb the stairs using the step-over-step method (where one foot is placed on each stair), without using the hand-rail, and only used data from the middle step. Any steps that did not meet these conditions were excluded from analysis. Five complete ascent and descent steps were used for analysis. We analysed the stance phase (defined from initial contact to lift-off of the supporting limb) and foot contact as when the vertical component of the ground reaction force exceeded 10 N.

### Outcome measures

For gait spatiotemporal outcomes were stride length, stride width, stride time and velocity. Kinematic and kinetic parameters analysed were restricted to the sagittal plane of the hip, knee, and ankle. We did not include other planes as we have found variability in those planes relatively high [20] and including these outcome measures would require an unrealistic sample size.

Data was low-pass filtered at 6 Hz using a Butterworth 4th order filter with zero phase lag. Joint angles and moments were calculated using Vicon Body Language software. For the gait task kinematic variables were normalised to percent gait cycle, kinematic variables normalised to percent stance phase. For the stair climbing tasks both kinematic and kinetic parameters were normalised to percentage stance phase.

### Statistical analysis

**Fig. 1:**
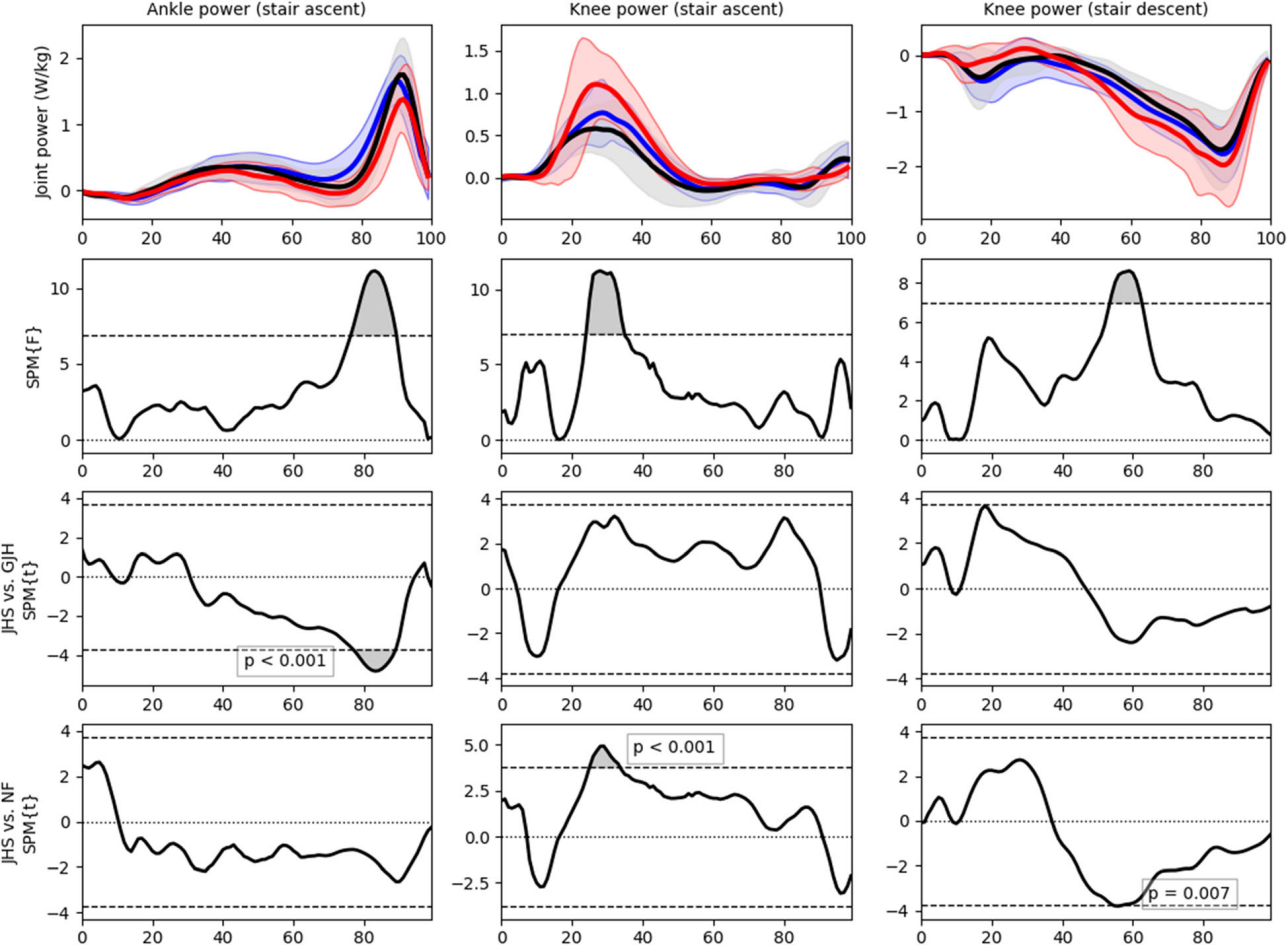
A selection of key SPM results. Top row show mean JHS (red), GJH (blue), and NF (black) joint powers. Second row shows the corresponding SPM ANOVA F statistic (dashed line equivalent to α = 0.05). Third and fourth rows show post-hoc SPM T-test comparisons for people with JHS vs. people with GJH, and people with JHS vs. people with NF respectively (dashed line equivalent to α = 0.0169 to account for multiple comparisons). Figures for all comparisons made in this paper can be found in supplementary material

Statistical parametric mapping (SPM) [21, 22] was used to statistically compare sagittal plane joint angles, moments, and powers between groups. SPM has been widely adopted in the field of biomechanics to investigate spatiotemporally continuous data. SPM was originally adopted in the field of neuro-imaging, and is especially applicable to movement data in that the data is bounded in time and/or space [22]. Here, a SPM one-way ANOVA was performed to examine the effect of group on each kinematic and kinetic variable; the F-statistic (SPM {F}) was calculated at each point of the time-series. Where SPM {F} crossed a threshold equivalent to α = 0.05 (above which only 5% of equally smooth random data would be expected to cross) post-hoc analysis was performed using SPM unpaired t-tests. For these post-hoc comparisons, the SPM{t} statistic was calculated for each between-group comparison. The critical threshold was set equivalent to α = 0.0169 to account for multiple comparisons. Significant differences were recorded where the SPM{t} crossed this threshold. See Fig. 1 for an example of the analysis process. The SPM analyses were conducted using open-source code (version 0.4.3, www.spm1d.org) in Python. Due to the large number of comparisons performed in the process of analysing multiple variables for multiple tasks, a selection of figures is presented in the main document. All figures are contained in the supplementary information section.

## Results

NF had a significantly lower Beighton score than JHS and GJH (*p* < .001 for both comparisons) groups; there was no significant difference between JHS and GJH (*p* = 1.000). The JHS group had a significantly greater VAS score than GJH and NF (p < .001 for both comparisons), and there was no significant difference between GJH and NF (p = 1.000). All participants completed the gait task but two JHS participants could not complete the stair task (one used the step-by-step method, and another was unable to perform the exercise without using the hand rails). Both participants were excluded from the analysis. The omissions did not alter the above significant differences between groups.

### Gait activity

Table 2 shows the spatiotemporal outcome measure results. Stride time was significantly longer in JHS than NF and GJH (*p* = .002 and *p* = .001 respectively). Gait velocity was significantly slower in JHS than NF () and GJH (*p* = .006 and *p* < .001 respectively). The GJH group had significantly greater stride length than NF and JHS (*p* < 0.001 in both cases).

**Table 2:** Results for gait

	JHS (*n*=23 for gait activity, *n*=21 for stair climbing activities)	GJH (*n*=23 in all activities)	NF (*n*=22 in all activities)
**Gait activity**			
Temporal spatial			
Stride time mean (s)^ab^	1.21±0.10	1.11±0.07	1.11±0.10
Velocity mean (m/s)^ab^	1.01±0.12	1.2±0.15	1.14±0.13
Stride width mean (m)	0.09±0.02	0.08±0.02	0.08±0.02
Stride length mean (m)^cb^	0.73±0.04	0.79±0.06	0.74±0.04
**Stair ascent activity**			
Stride Width (cm)	31.94 ± 10.58	31.35 ± 9.29	29.43 ± 11.28
Stance Time (s)^ab^	1.36 ± 0.71	0.89 ± 0.13	0.94 ± 0.1
Velocity (cm/s)	2.6 ± 0.9	3.8 ± 1.7	2.7 ± 1.2
**Stair descent activity**			
Stride Width (cm)^b^	25.71 ± 2.88	23.94 ± 1.56	25.21 ± 2
Stance Time (s)^ab^	1.06 ± 0.26	0.77 ± 0.1	0.82 ± 0.1
Velocity (cm/s)	4.1 ± 2.6	3.3 ± 1.7	3.1 ± 1.8

^a^ significant difference between JHS and NF groups, ^a^significant difference between JHS and GJH groups, ^c^ significant difference between NF and GJH groups

#### Moving to the SPM analysis

Mean kinematic variables were highly similar for the majority of the gait cycle (see figure in supplementary material). The SPM ANOVA indicated that there were significant differences at each joint, however in post-hoc pairwise comparisons the critical threshold was not exceeded at any joint (see figures in supplementary material).

For joint moments the SPM ANOVA indicated there was a significant difference between groups at the ankle and the hip. Investigating these comparisons further; post-hoc SPM t-tests indicated that JHS had a significantly lower ankle moment than both GJH and NF between ~ 80% and ~ 90% of stance phase (*p* = 0.009 and *p* = 0.001 respectively). At the hip JHS had a significantly smaller flexor moment than NF at 0% to ~ 5% of stance phase (*p* = 0.013), and significantly smaller extensor moment than GJH (*p* < 0.001) and NF (p < 0.001) at ~ 70% to ~ 100% stance phase.

For joint powers the SPM ANOVA indicated there was a significant difference between groups at the ankle and the hip. At the ankle JHS had significantly lower power generation between ~ 90% to ~ 95% of stance phase than both GJH (*p* = 0.001) and NF (*p* = 0.002). At the hip JHS had lower power absorption at ~ 75% of stance phase than GJH (*p* = 0.010) and NF (*p* = 0.017). Toward the end of stance phase at ~ 95 to 100%, JHS had significantly lower power generation than NF (*p* = 0.005).

### Stair ascent

For spatiotemporal measures, the only significant difference recorded between groups was in stance time; JHS had a significantly longer stance time than both GJH and NF groups (*p* < .001 in both cases).

For joint angle, the SPM ANOVA indicated there was a significant difference between groups at the hip. Post-hoc tests indicated that JHS had greater knee flexion at 0% to ~ 30% of the gait cycle than NF (*p* = 0.002).

For joint moments, the SPM ANOVA indicated there was a significant difference between groups at the ankle and knee. At the ankle JHS plantarflexor moment was lower than both GJH and NF groups for the entire stance phase. This difference reached significance early in stance phase, at ~ 0% compared to GJH (*p* = 0.017) and ~ 5 to 30% compared to NF (*p* < 0.001); and later in stance phase, ~ 75% to ~ 90% compared to GJH (p < 0.001), and ~ 90% compared to NF. At the knee the JHS had a significantly smaller extension moment then GJH at ~ 0% stance phase (p = 0.017) and a smaller extension moment than NF ~ 80% to ~ 95% stance phase (*p* < 0.001).

For joint powers, the SPM ANOVA indicated there was a significant difference between groups at the ankle and knee. JHS generated significantly less power at the ankle than GJH at ~ 75% to ~ 90% of stance phase (p < 0.001). At the knee, JHS generated significantly more power the NF at ~ 25% to ~ 35% stance phase (p < 0.001).

### Stair descent

For temporospatial measures, JHS had a significantly longer stance duration than GJH and NF (*p* < .001 in both cases). JHS had a significantly wider stride width than GJH (*p* = .038) but not NF. There was no significant difference between groups for velocity.

Moving to the SPM results for joint angle. The SPM ANOVA indicated a significant difference between groups for ankle and hip angles. Post-hoc SPM t-tests for ankle angle did not reveal any significant differences. At the hip, mean joint angle for the JHS group was more flexed for all of stance phase, this reached significance when comparing to NF at 0% to ~ 80% of stance phase (*p* < 0.001).

For joint moment, the SPM ANOVA indicated a significant difference between groups in hip moment. Post-hoc SPM t-tests showed that JHS had a greater hip flexor moment than GJH at ~ 10% to ~ 30% of stance phase (p < 0.001) and greater than NF at ~ 15% stance phase (*p* = 0.015).

For joint power, the SPM ANOVA indicated a significant difference between groups at the knee. Post-hoc SPM t-tests indicated this difference was significant at one small cluster at ~ 55% of stance phase, where JHS had significantly lower power absorption than NF (*p* = 0.007).

## Discussion

This study was novel in including both people with GJH and people with NF as control groups. Our aim was to show if it is the hypermobility causing differences in movement or another factor of JHS. Across all activities in this study, for both temporospatial and SPM outcome measures, there was only one significant difference between GJH and NF groups (stride length during gait). In comparison there were many significant differences when comparing people with JHS and people with GJH, and when comparing people with JHS to people with NF. Although there was only one significant difference between people with GJH and NF, visual inspection of the kinematic, moment, and power figures (see Fig. 1 and supplementary material) show that people with GJH commonly fall ‘between’ the NF and JHS groups. This could hint that hypermobility partially contributes to differences in movement. However, since there were no actual significant differences between people with GJH and people with NF we conclude that other feature(s) of JHS contribute more in causing functional impairments. The multifactorial nature of JHS means that there are many potential factors that could cause, or contribute to, the differences observed in this study.

Before discussing the specific differences between groups, it is important to note that our participants included more women than men; 87% of our JHS group were female. JHS and GJH are more common in females [23], and a recent study of a Wales population found that of people diagnosed with HSD or EDS, 70% were female [24]. With this in mind, we think that these results are generalisable to the JHS patient cohort.

Our objective was to identify differences in any functional impairments during gait and stair-climbing. For the gait activity the JHS group had a slower stride time, greater mean stride width, lower velocity, and smaller stride length than NF and GJH groups (of which velocity and stride time were significant). These results build on other studies of JHS gait such as Alsiri *et al*^14^*.* and indicate that the JHS group had impaired gait function. In terms of kinematics, there were no significant differences between groups for sagittal plane joint angle. This is interesting as recently Alsiri et al.^14^ found that during gait people with JHS had reduced peak knee flexion compared to people with NF. Peak knee flexion occurs in swing phase to provide limb clearance for the swing limb and can be reduced by pain, muscle weakness, and lower walking velocity [25, 26]. It is therefore surprising that we did not observe this in this study, despite all individuals with JHS having knee pain and people with JHS generally suffering from muscle weakness [27, 28]. Although JHS did not show reduced peak knee flexion compared to NF, JHS did show a significantly lower peak hip extensor moment than NF and GJH, and reduced hip moments during gait have been associated with reduced peak knee flexion [29]. A potential reason for the different findings between this study and other studies of gait in people with JHS is differences in methodology. For example, to our knowledge this is the first study to use SPM to investigate biomechanics of people with hypermobility during gait and stair climbing.

Although people with JHS showed lower hip extension throughout the gait cycle, this difference did not reach significance (see supplementary material for a figure). The SPM did show significant differences in hip joint moment and power between groups; people with JHS showed reduced flexor moment in early stance and reduced extensor moment in late stance. This could be caused by people with JHS having hip weakness. One function of the hip extensor moment is to stabilise the trunk from flexing forward during the first half of stance phase [30], since the moment is reduced it is feasible that the trunk is being allowed to flex forward, a per the mechanism to stabilise the knee and aid pull-off [31]. Equally however, the reduction in hip moment could show a reluctance to generate muscular force due to pain. The lower hip extensor moment in late stance corresponds to the JHS group showing significantly lower power generation at the hip in late stance. This is accompanied by people with JHS showing significantly lower power generation than both control groups at the ankle in late stance, and people with JHS showing greater power generation at the knee in late stance than people with NF. It is interesting that despite all people with JHS in this study having knee pain, the results indicate that their gait strategy is reducing moments and powers at the ankle and hip in favour of generating power at the knee. A potential reason for this could be people with JHS adapting to weakness and/or instability; reduced peak hip extension is commonly seen in deficient gait, and could be due to muscle weakness of the hip flexors [32]. Hip extension can also be reduced by forward trunk lean; tilting the pelvis forward anteriorly reduces hip flexor work by negating the effort used to support the trunk [31]. Flexing the trunk also moves the body’s centre of gravity/ground reaction line anteriorly relative to the knee joint, which stabilises the knee joint and aids hip flexion, which aids the pull-off of the limb during pre-swing [31]. Although the position of the trunk was not measured in this study, this adaption has been seen in stair climbing of GJH individuals [33]. Interestingly, the significant differences in power peaks were all found in late stance phase, the late-stage peaks are where the body generates energy to propel the body forward, and muscle weakness of JHS could be limiting the amount of power produced.

We included stair-climbing in this study as it is an activity that places greater demands on the body than level walking and might highlight functional impairments. In both stair ascent and descent, stride time was significantly slower in JHS than GJH and NF groups, whereas there was no significant difference between GJH and NF groups. Speed of the movement influences the demand on muscles to decelerate and accelerate the centre of mass [34]. Muscle activity, and possibly pain, can be lessened by reducing the demand on the muscles by using a slower movement and/or a limited range of motion [34]. This suggests that the activity of stair climbing is impaired in JHS. In conditions such as total knee arthroplasty, decreased muscle strength was found to lower gait velocity during stair descent [35]. This is feasibly the reason here, as people with JHS do tend to have reduced muscle strength [27, 28]. Although other factors of JHS such as fatigue, joint pain, and impaired balance cannot be disregarded.

A reduction in knee flexion during stair ascent has been observed in other cohorts who have knee pain [34, 36], here JHS knee flexion was not significantly different to controls, which suggests that this compensatory mechanism is not being used. The only significant difference observed in kinematics between groups was mean hip flexion; people with JHS were more flexed than people with NF during early stance. As reported for gait, this could represent forward trunk lean, which lowers the demand on the knee extensors [37] and may be a strategy to reduce knee pain or compensate for muscle weakness in JHS participants.

In terms of joint moments and powers, a similar strategy was observed in people with JHS to the gait activity. People with JHS had significantly lower plantarflexor moment that both control groups in early and late stance phase, and significantly lower power generation at the ankle than people with GJH in late stance. Although people with JHS showed a significantly lower knee extensor moment in late stance than people with NF, during early stance they had significantly higher power generation at the knee. Taken as a whole, this shows that the people with JHS were favouring a knee strategy to ascend stairs. Wilken et al. [38] noted that although knee power is responsible for generating a significant proportion of positive power during stair ascent, the majority of power is produced while the acceleration of the centre of mass is near zero. The authors stipulate that the ankle is used to initiate acceleration of the centre of mass, and the knee is responsible for maintaining vertical velocity of the body. It seems feasible that people with JHS are using more of a “knee-strategy” to produce the force necessary to raise the centre of mass, with a reluctance to use the ankle. This potential reluctance to use the ankle is reflected in the significantly lower power generation people with JHS group exhibited during late the push-off phase of late stance. Therefore, if the ankle is exhibiting lower power, the knee produces relatively more force than normal. Consequently, the increased hip flexion (and inferred trunk lean), may be a compensatory strategy to limit the extra forces at the knee; i.e. impairments in the distal lower limb joints being adapted for by more proximal joints. The fact that there were no significant differences between GJH and NF groups, for joint angle, moment, or power parameters, would indicate that people with asymptomatic joint hypermobility are able to use the ‘normal’ ankle strategy to provide vertical acceleration during stair ascent.

During stair descent people with JHS again showed increased hip flexion; mean hip angle was more flexed than people with GJH and people with NF for all of stance phase, and this difference was significant between NF and JHS for the majority of stance phase. Increased hip flexion may be associated with forward trunk lean; a mechanism described previously to reduce moment and contact force at the knee. Interestingly this increased flexion corresponds to a reversal in hip joint moment in early stance; people with JHS showed small flexor moment in early stance, whereas there was an average extensor moment for people with GJH and people with NF. Hip moments during stair climbing can be highly variable between individuals due to the variety of trunk positions assumed [16]. In common with gait and stair-ascent activities, these results hint that people with JHS favour a knee-strategy; the JHS group showed significantly more power absorption during mid stance-phase than people with NF (see Fig. 1 and supplementary material).

A limitation of this study is that muscle strength was not measured, and in general we are not able to determine whether the differences in movement lead to symptoms or whether the symptoms lead to the differences in movement. Irrespective of whether these differences are cause or effect, the benefit of future work would be to see if increasing muscle strength of JHS individuals leads to a normalisation of kinematic and kinetic parameters and a reduction in pain. This study was limited to sagittal plane motion only. It may be clinically relevant to understand potential differences in other planes of motion. However, a priori work indicated that transverse and frontal plane movements were much more variable. Our aim is to enable clinicians to target treatment, therefore we did not include transverse and frontal planes since it is unlikely they could be reliably re-tested in future work, and secondly to keep the sample size manageable.

## Conclusions

Across both activities there were many significant differences between the group with JHS and control groups. In contrast, even though the GJH group were equally as hypermobile as the JHS group, only one significant difference was observed between GJH and NF groups. We therefore conclude that it is not hypermobility alone that is causing the observed differences, but rather other factors of JHS. GJH individuals may use compensatory mechanisms to ‘overcome’ their hypermobility, which JHS individuals perhaps do not employ. However, since NF and GJH groups were so similar across all outcome measures, it would appear that GJH gait is the same as NF gait, and such adaptations do not need to be made.

The second objective was to identify any potential impairments to direct treatment. Our interpretation of the results is that participants with JHS switch from generating force at the ankle, to generating force at the knee during stair ascent. Similar differences between JHS and NF /GJH groups can be observed during stair-descent. The differences in lower limb joint kinematics and kinetics observed in this study suggest treatment strategies to address these changes. For example, testing if physical therapy could normalise stair climbing, with a target of strengthening plantaflexors of the ankle in particular, so they can generate the force during stair ascent and focus on quadriceps strength to reduce the load through the ankle during stair descent.

## Supplementary Information

**Additional file 1.**

## Data Availability

The datasets used and/or analysed during the current study are available from the corresponding author on reasonable request.

## Abbreviations

GJH: Generalised Joint Hypermobility
HDCT: Heritable Disorder of Connective Tissue
JHS: Joint Hypermobility Syndrome
NF: Normal Flexibility
