# Supplementary material for: Comparing sagittal plane kinematics and kinetics of gait and stair climbing between hypermobile and non-hypermobile people; a cross-sectional study
Source: BMC Musculoskelet Disord. 2021 Aug 19;22:712. doi: 10.1186/s12891-021-04549-2 (PMC8377885; doi:10.1186/s12891-021-04549-2)
Supplement: Supplementary file 1 — Additional file 1. [file 12891_2021_4549_MOESM1_ESM.docx]

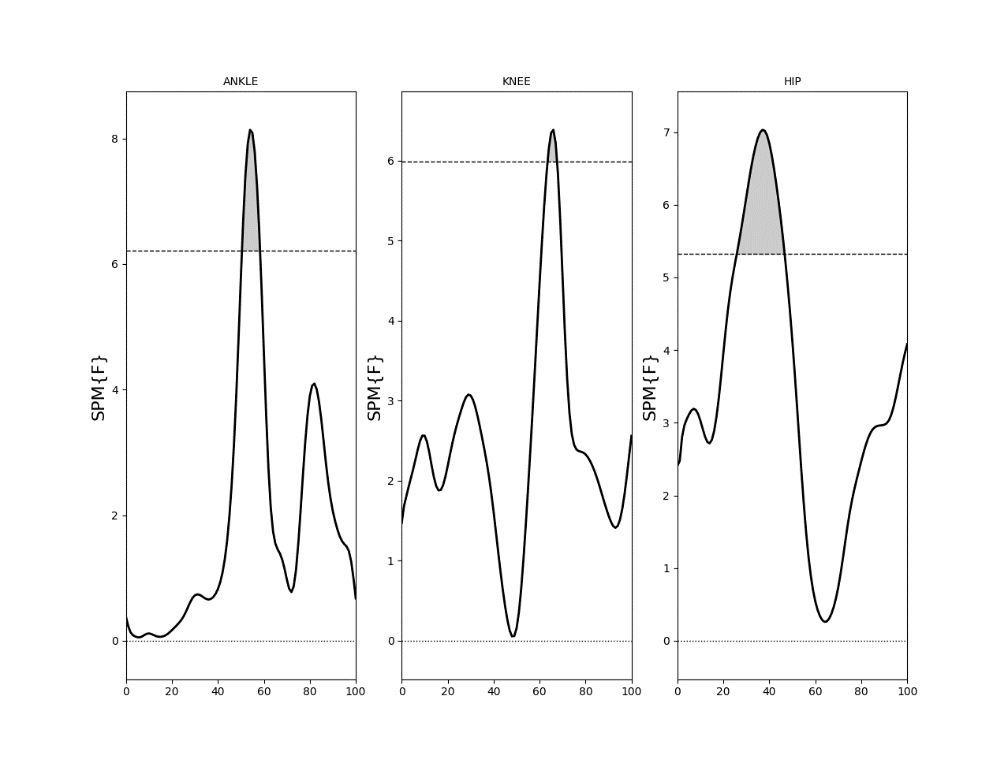


Supplementary figure 1: SPM ANOVA for the gait joint angle. Dashed line equivalent to α=0.05.


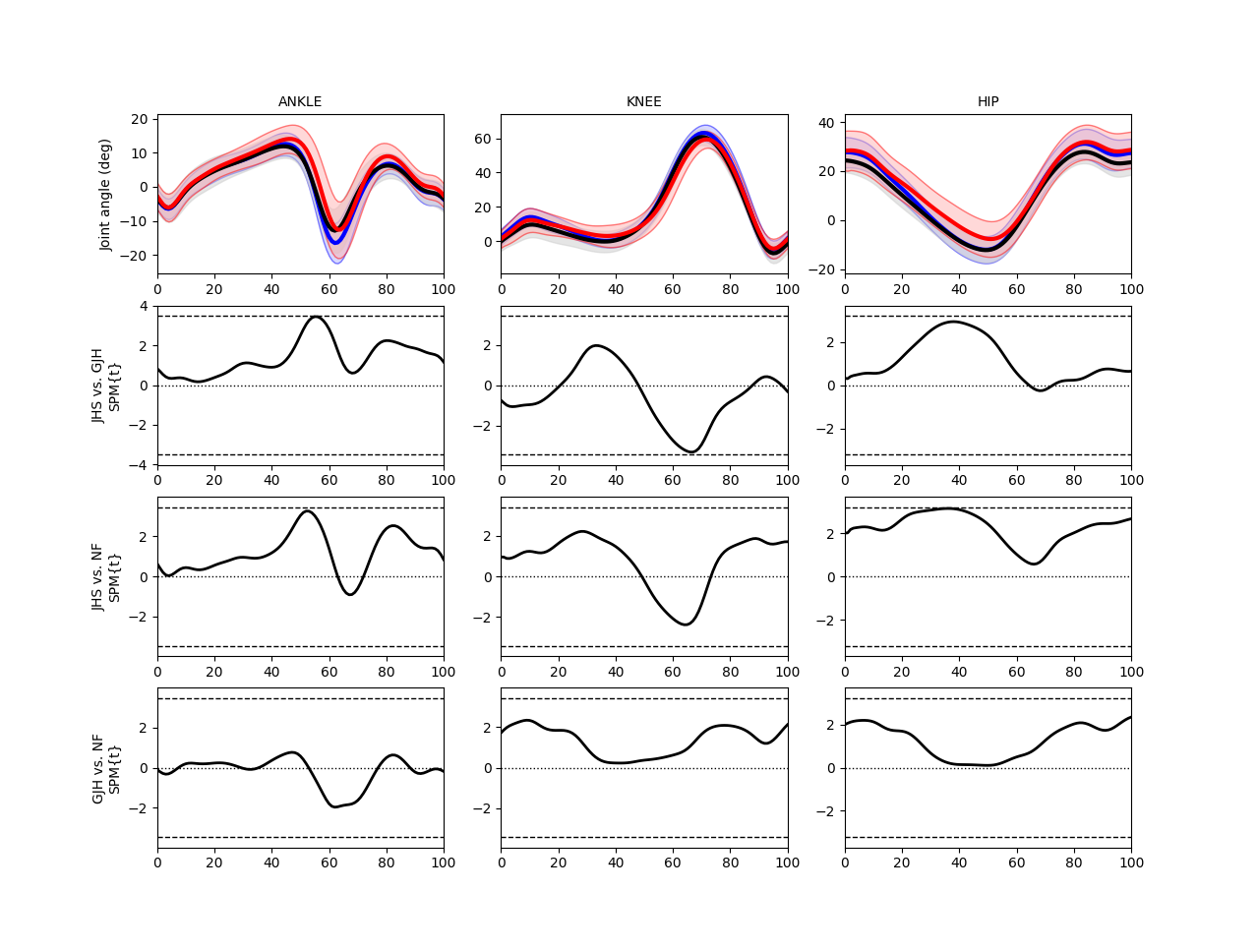


Supplementary figure 2. SPM post-hoc t-tests for the Gait joint angles. Horizontal axis is percent gait cycle. Top row is mean joint angle ± 1 standard deviation for people with JHS (red), people with GJH (blue), and NF (black). Second to fourth rows show SPM{T} value throughout the gait cycle. Dashed lines equivalent to α=0.0169.


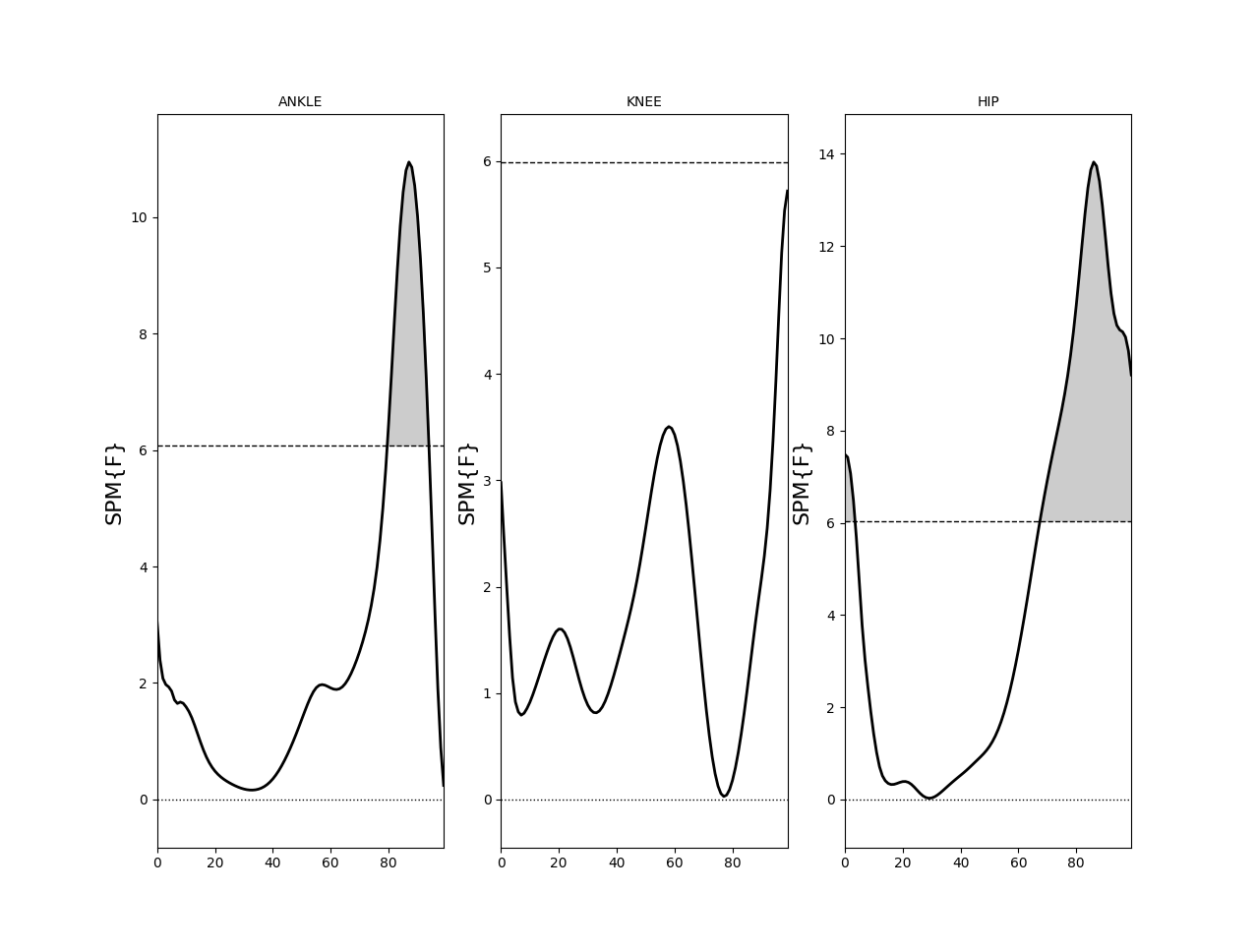


Supplementary figure 3: SPM ANOVA for the gait joint moment. Dashed line equivalent to α=0.05.


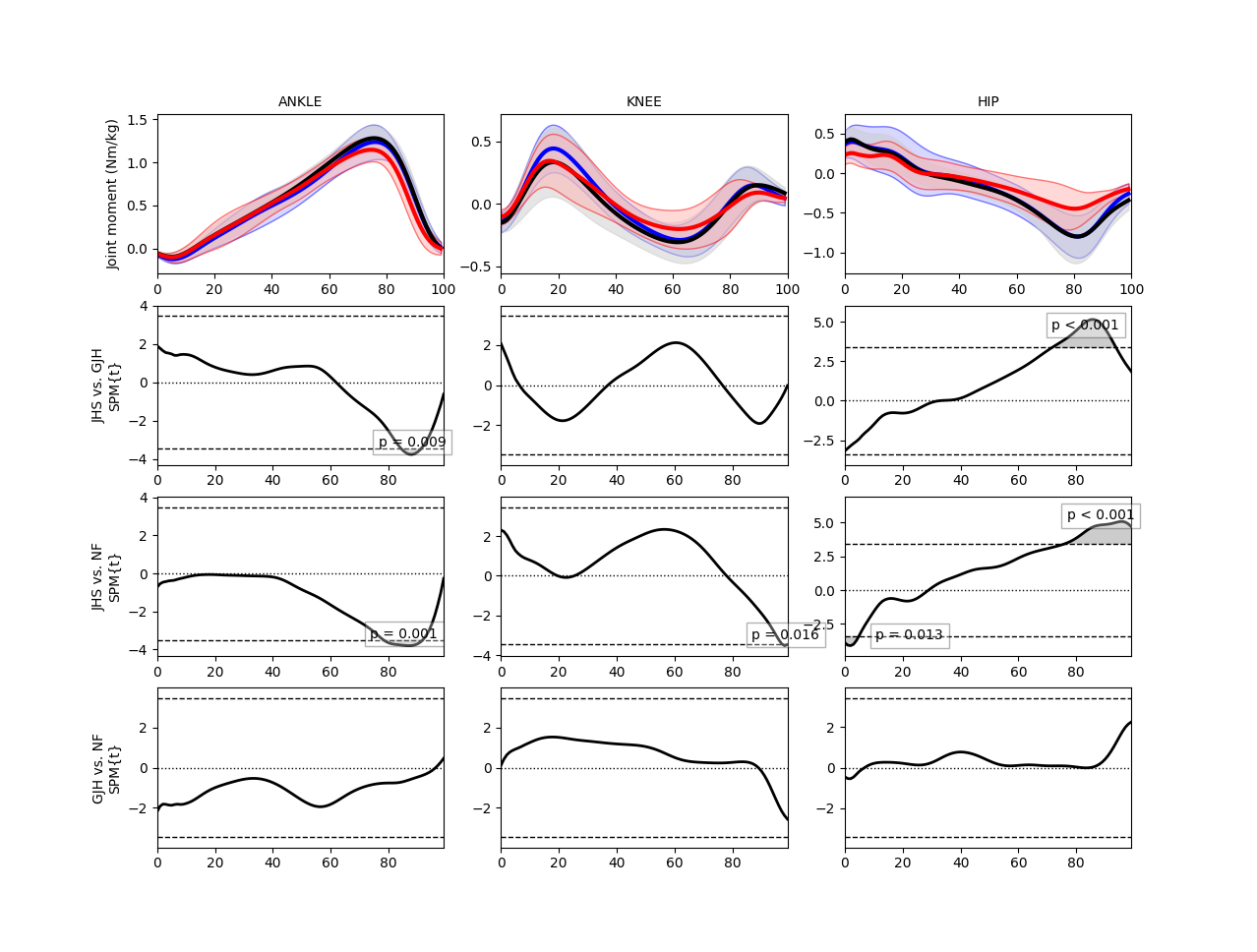


Supplementary figure 4. SPM post-hoc t-tests for Gait joint moment. Horizontal axis is percent stance phase. Top row is mean joint moment ± 1 standard deviation for people with JHS (red), people with GJH (blue), and NF (black). Second to fourth rows show SPM{T} value throughout stance phase. Dashed lines equivalent to α=0.0169.


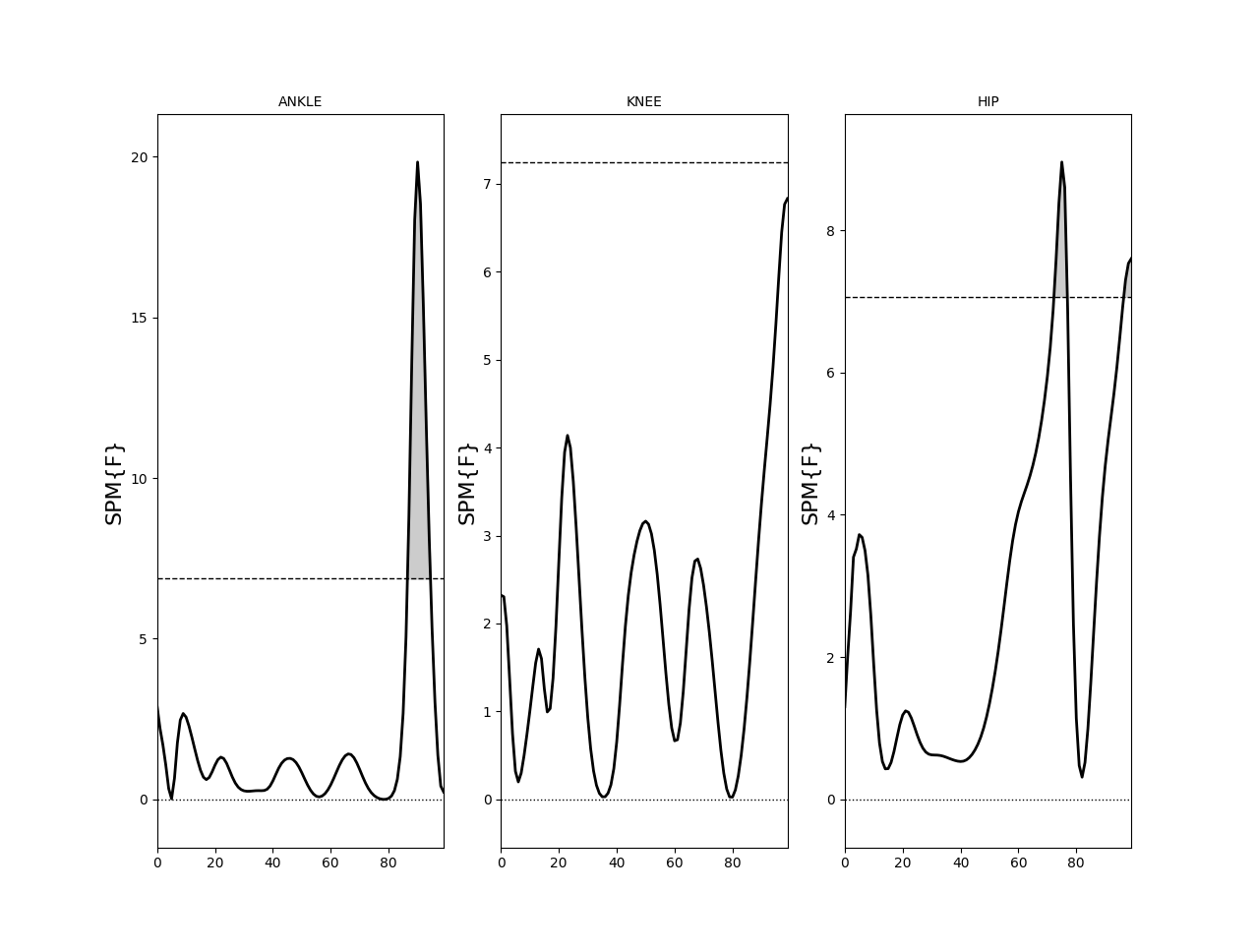


Supplementary figure 5: SPM ANOVA for the gait joint power. Dashed line equivalent to α=0.05.


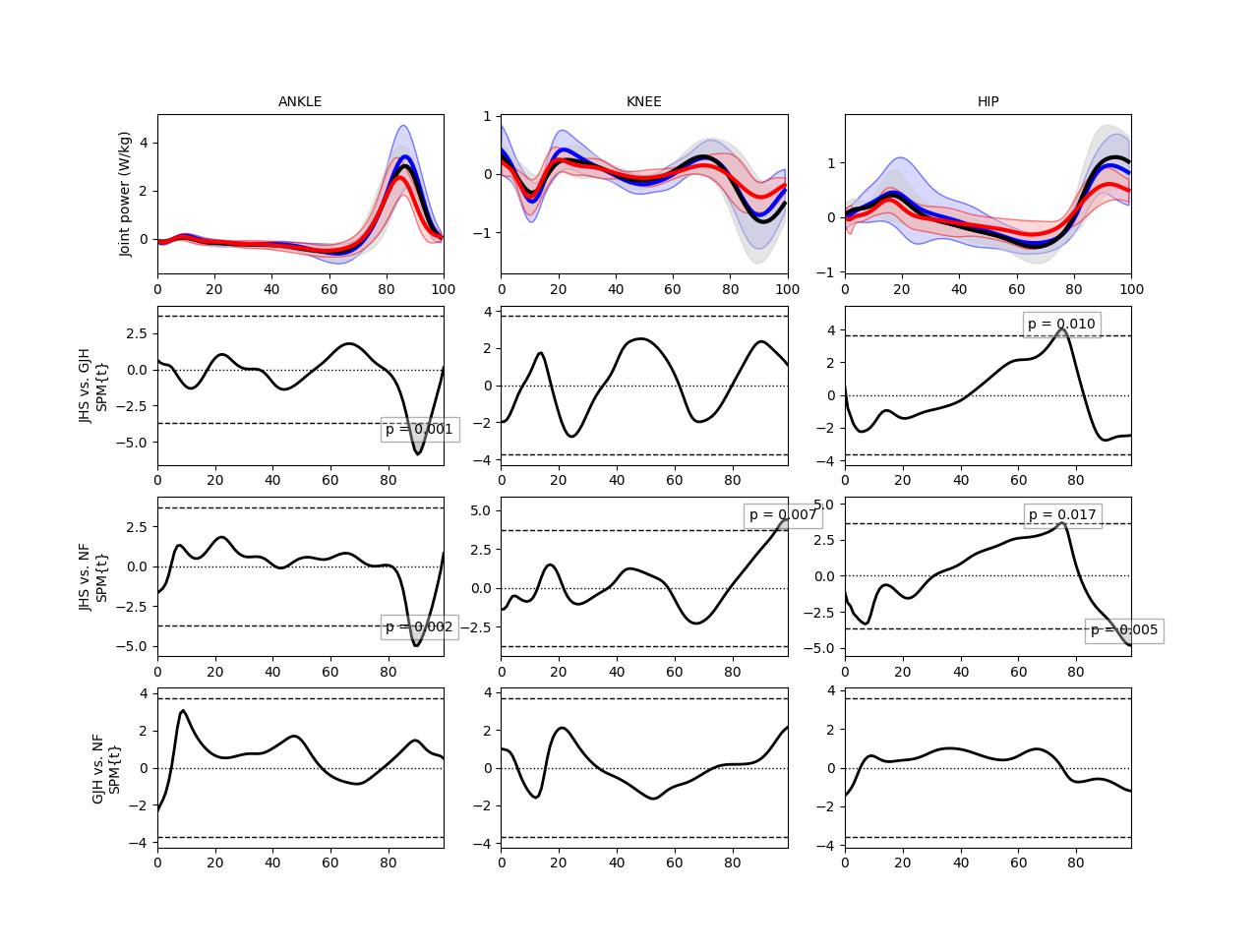


Supplementary figure 6. SPM post-hoc t-tests for Gait joint power. Horizontal axis is percent stance phase. Top row is mean joint power ± 1 standard deviation for people with JHS (red), people with GJH (blue), and NF (black). Second to fourth rows show SPM{T} value throughout stance phase. Dashed lines equivalent to α=0.0169.


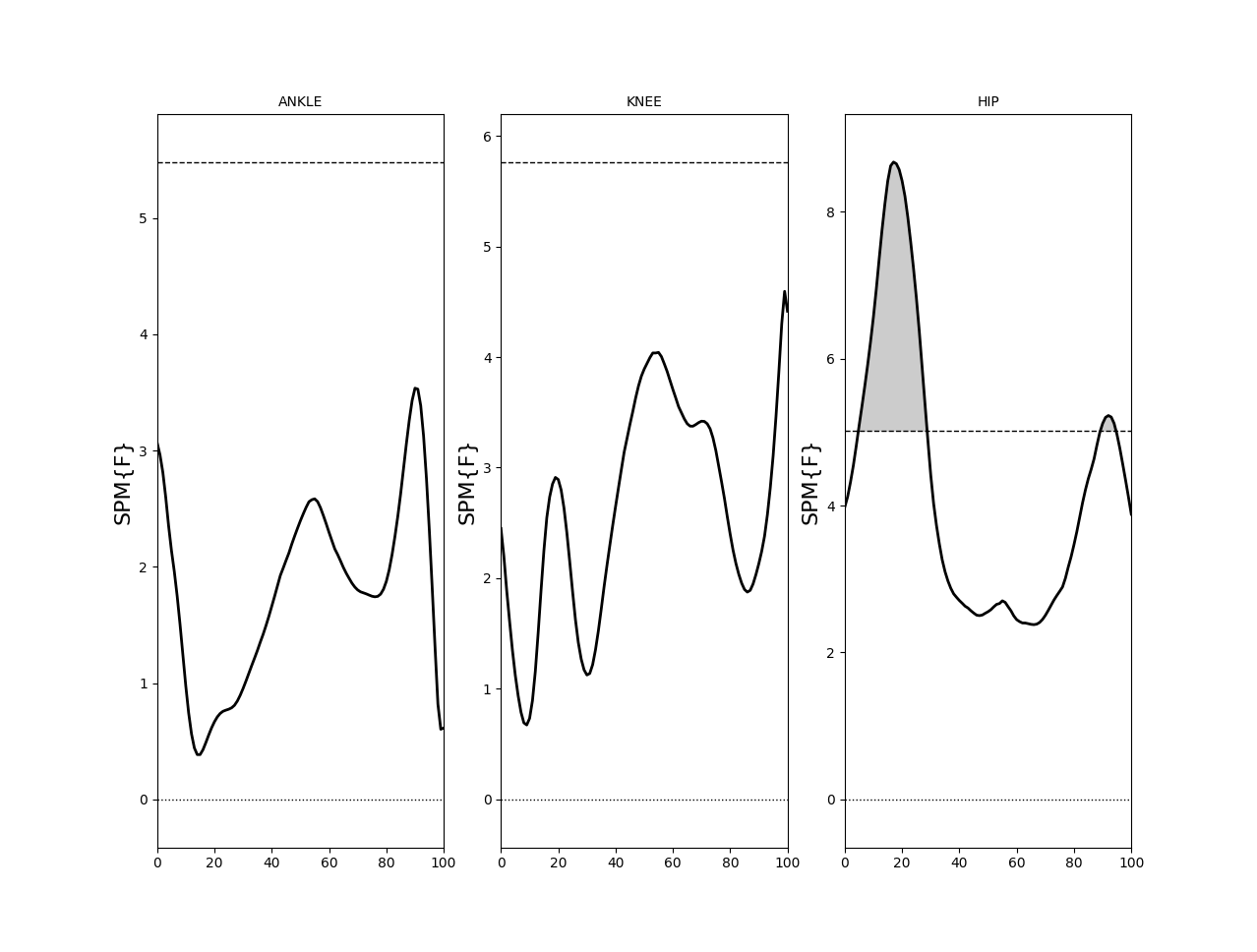


Supplementary figure 7: SPM ANOVA for the stair ascent joint angle. Dashed line equivalent to α=0.05.


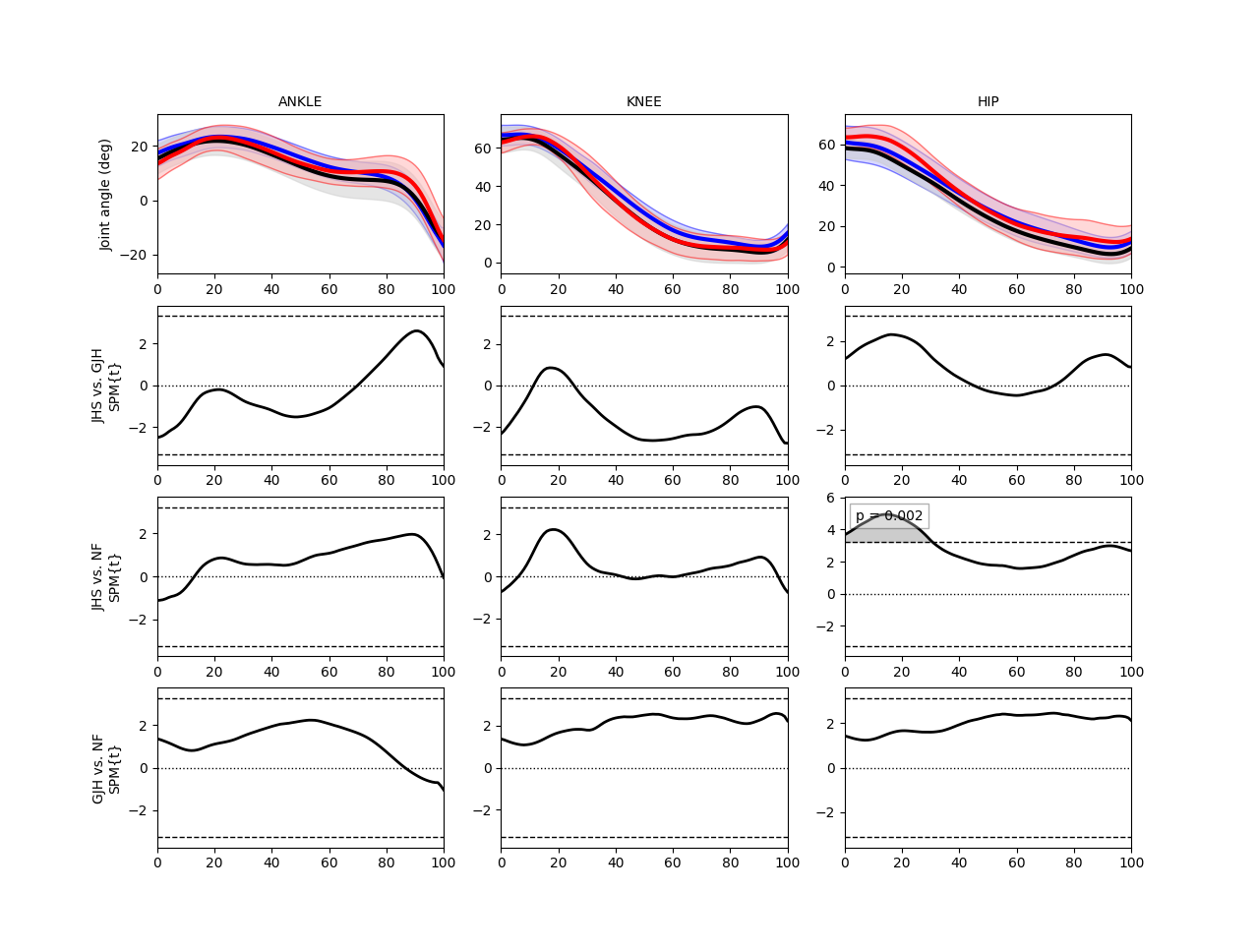


Supplementary figure 8. SPM post-hoc t-tests for stair ascent joint angle. Horizontal axis is percent stance phase. Top row is mean joint angle ± 1 standard deviation for people with JHS (red), people with GJH (blue), and NF (black). Second to fourth rows show SPM{T} value throughout stance phase. Dashed lines equivalent to α=0.0169.


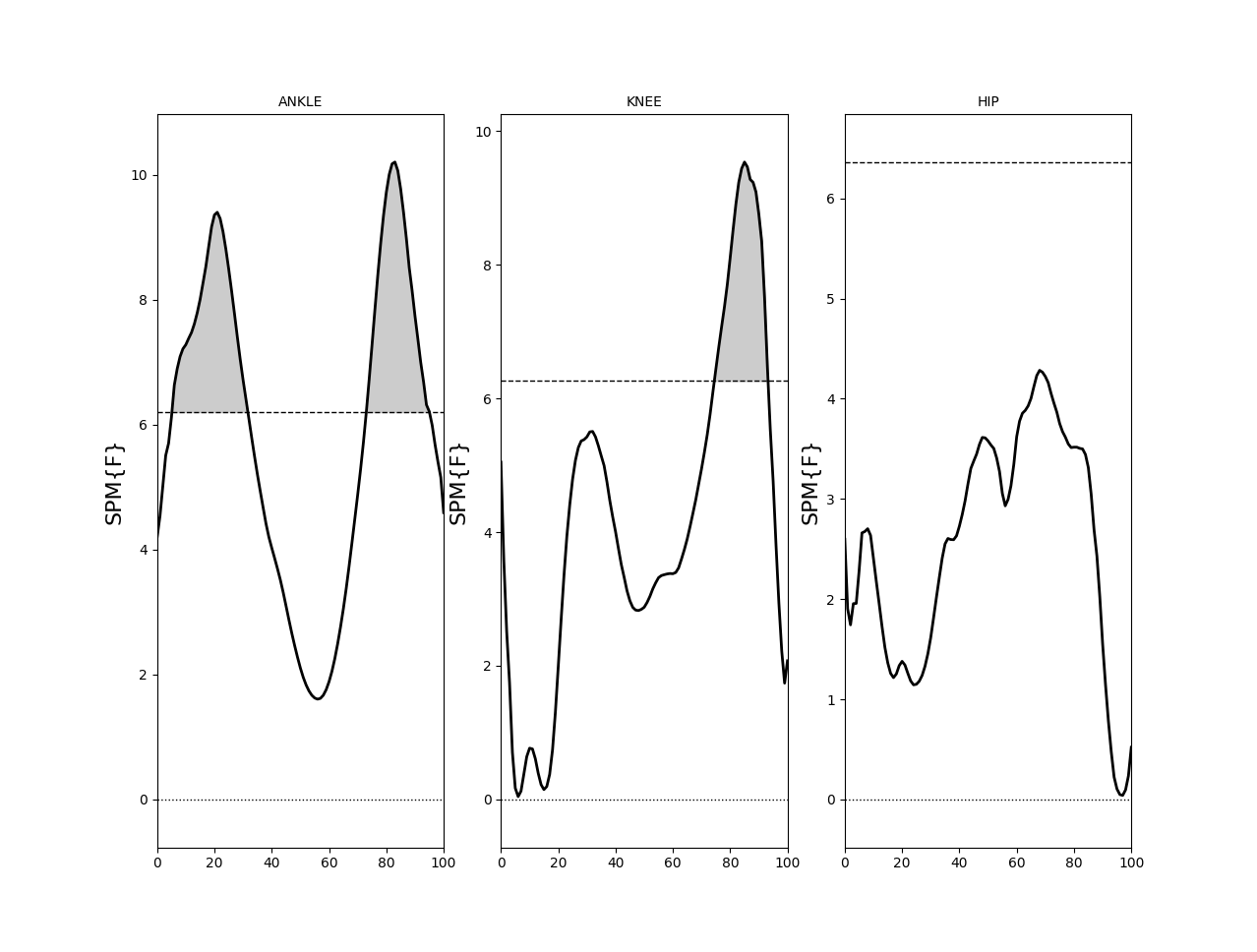


Supplementary figure 9: SPM ANOVA for the stair ascent joint moment. Dashed line equivalent to α=0.05.


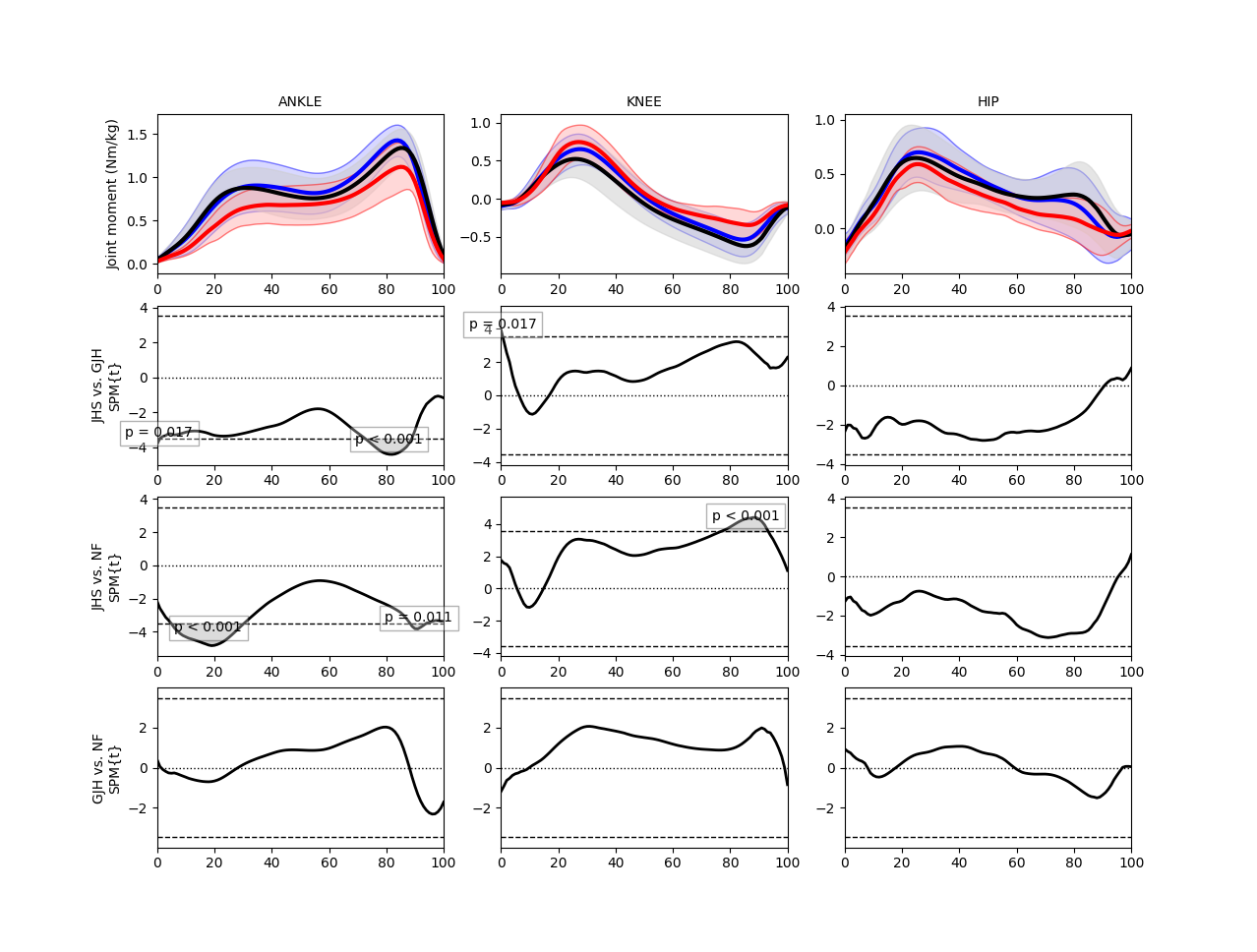


Supplementary figure 10. SPM post-hoc t-tests for stair ascent joint moment. Horizontal axis is percent stance phase. Top row is mean joint moment ± 1 standard deviation for people with JHS (red), people with GJH (blue), and NF (black). Second to fourth rows show SPM{T} value throughout stance phase. Dashed lines equivalent to α=0.0169.


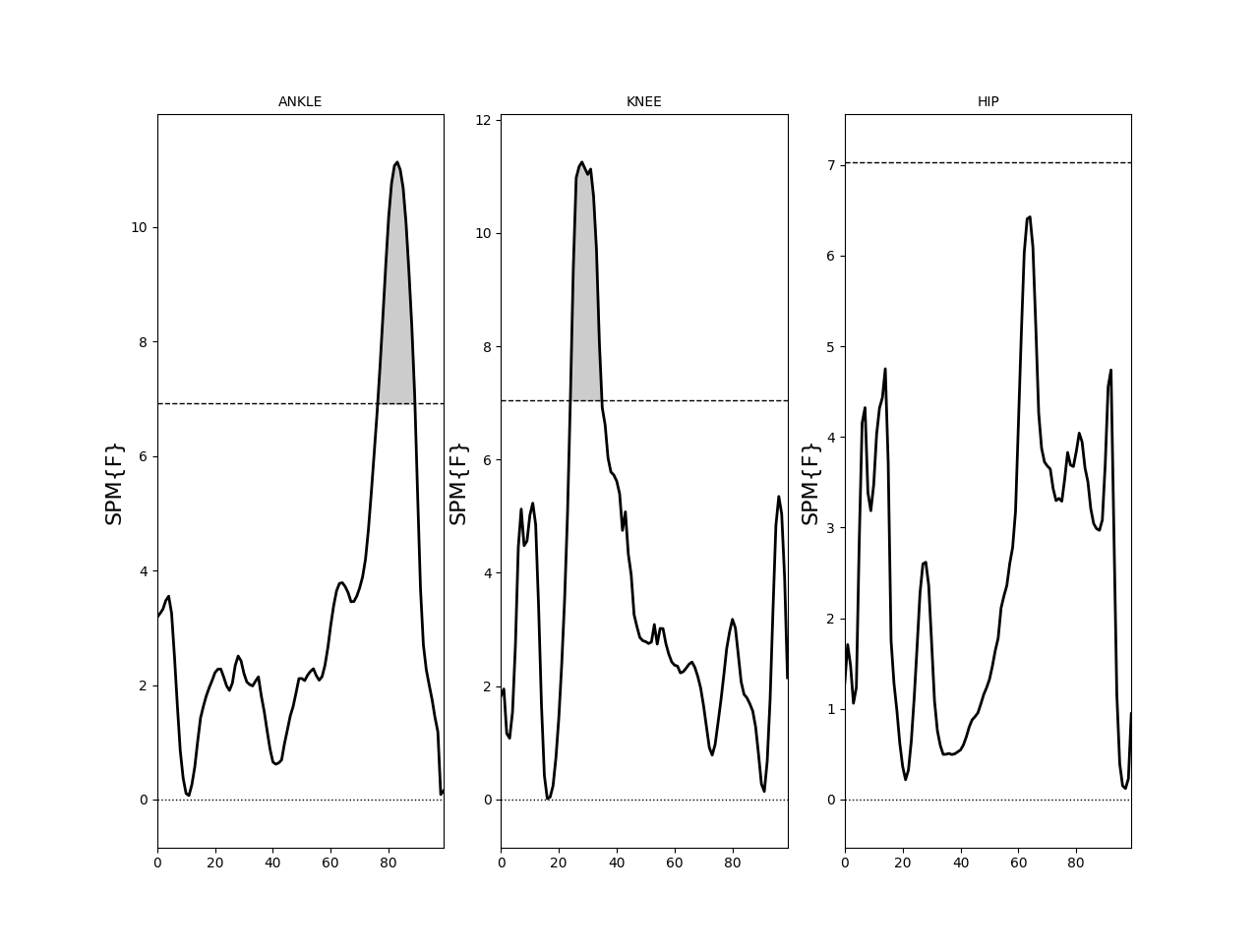


Supplementary figure 11: SPM ANOVA for the stair ascent joint power. Dashed line equivalent to α=0.05.


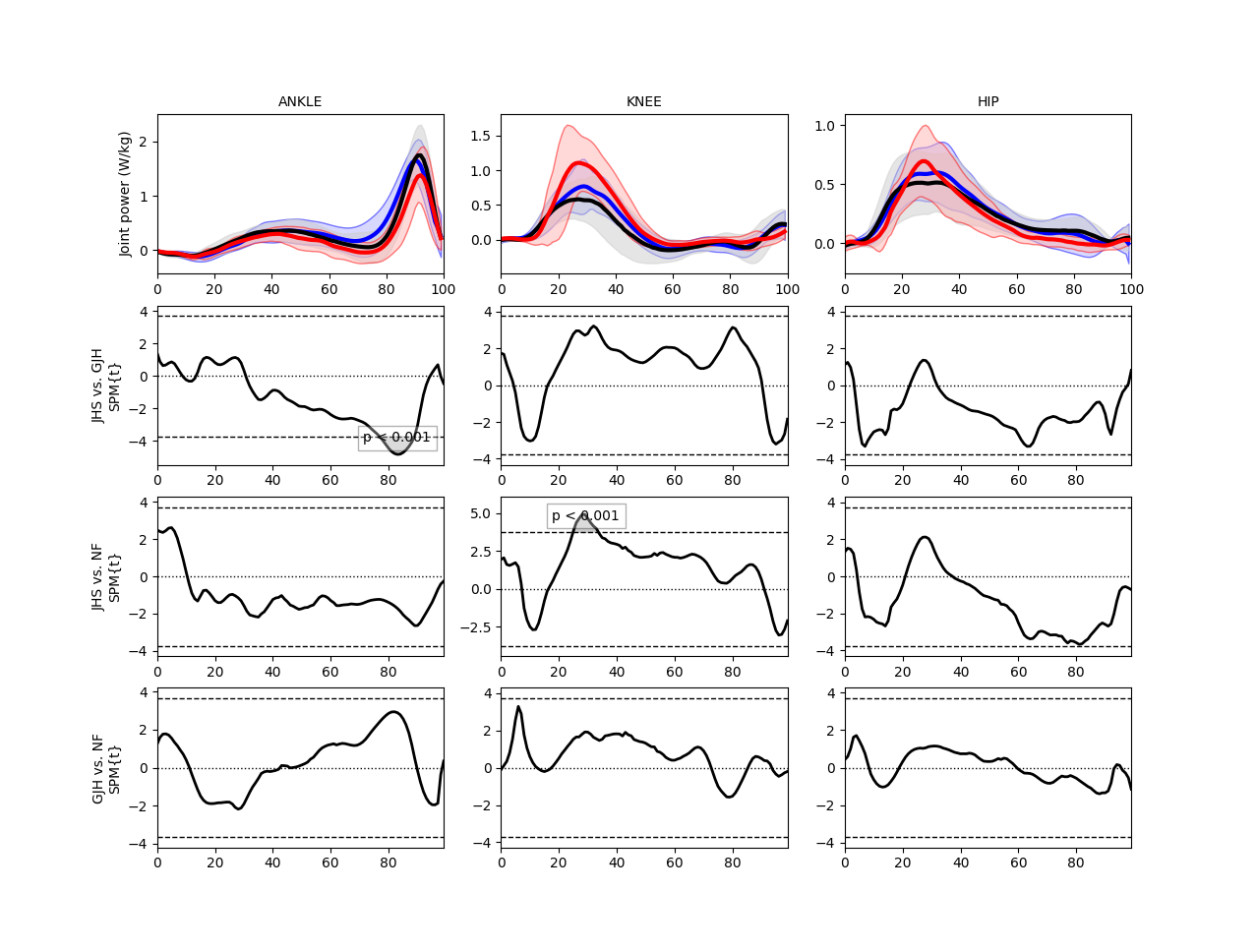


Supplementary figure 12. SPM post-hoc t-tests for stair ascent joint power. Horizontal axis is percent stance phase. Top row is mean joint power ± 1 standard deviation for people with JHS (red), people with GJH (blue), and NF (black). Second to fourth rows show SPM{T} value throughout stance phase. Dashed lines equivalent to α=0.0169.


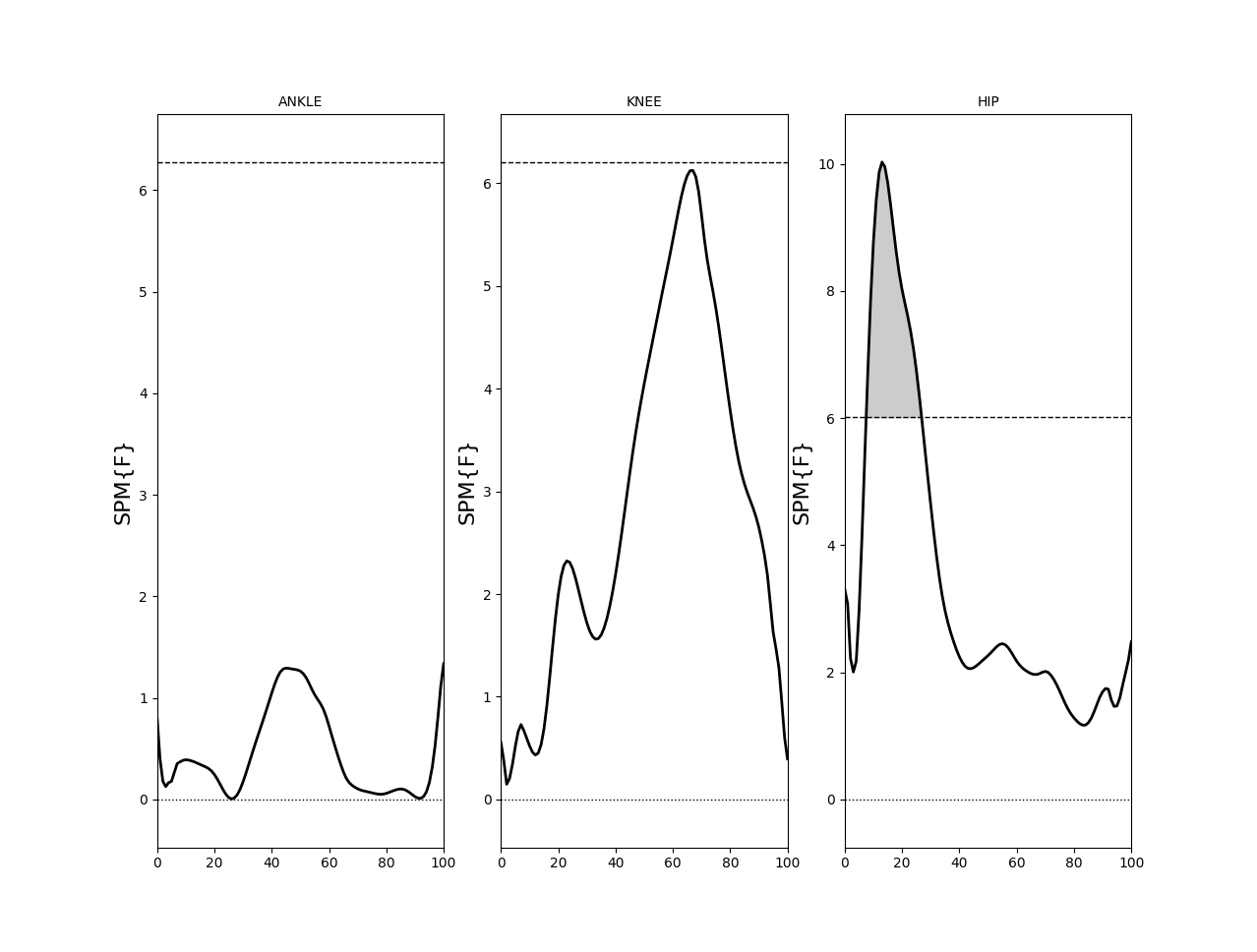


Supplementary figure 13: SPM ANOVA for the stair descent joint angle. Dashed line equivalent to α=0.05.


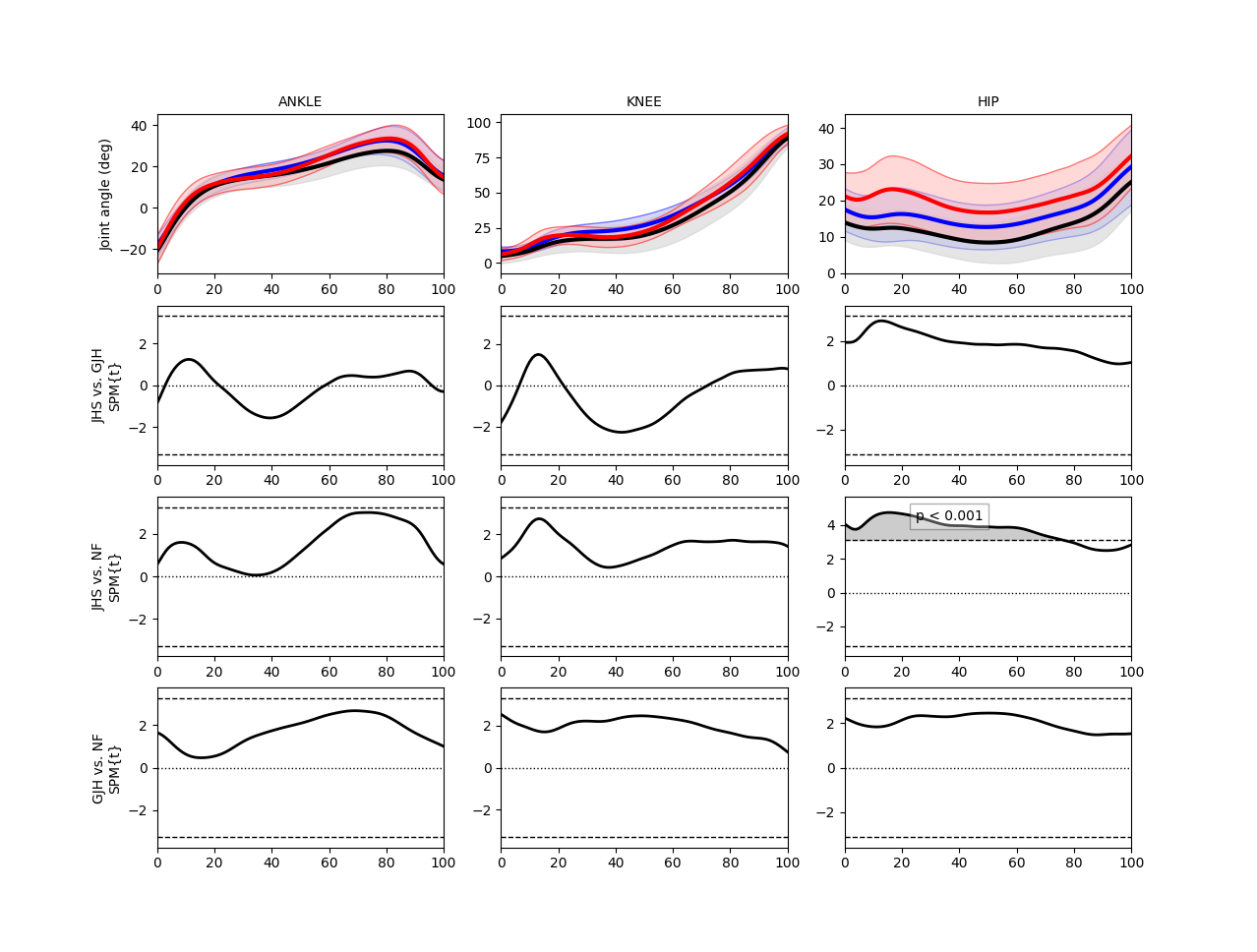


Supplementary figure 14. SPM post-hoc t-tests for stair descent joint angle. Horizontal axis is percent stance phase. Top row is mean joint angle ± 1 standard deviation for people with JHS (red), people with GJH (blue), and NF (black). Second to fourth rows show SPM{T} value throughout stance phase. Dashed lines equivalent to α=0.0169.


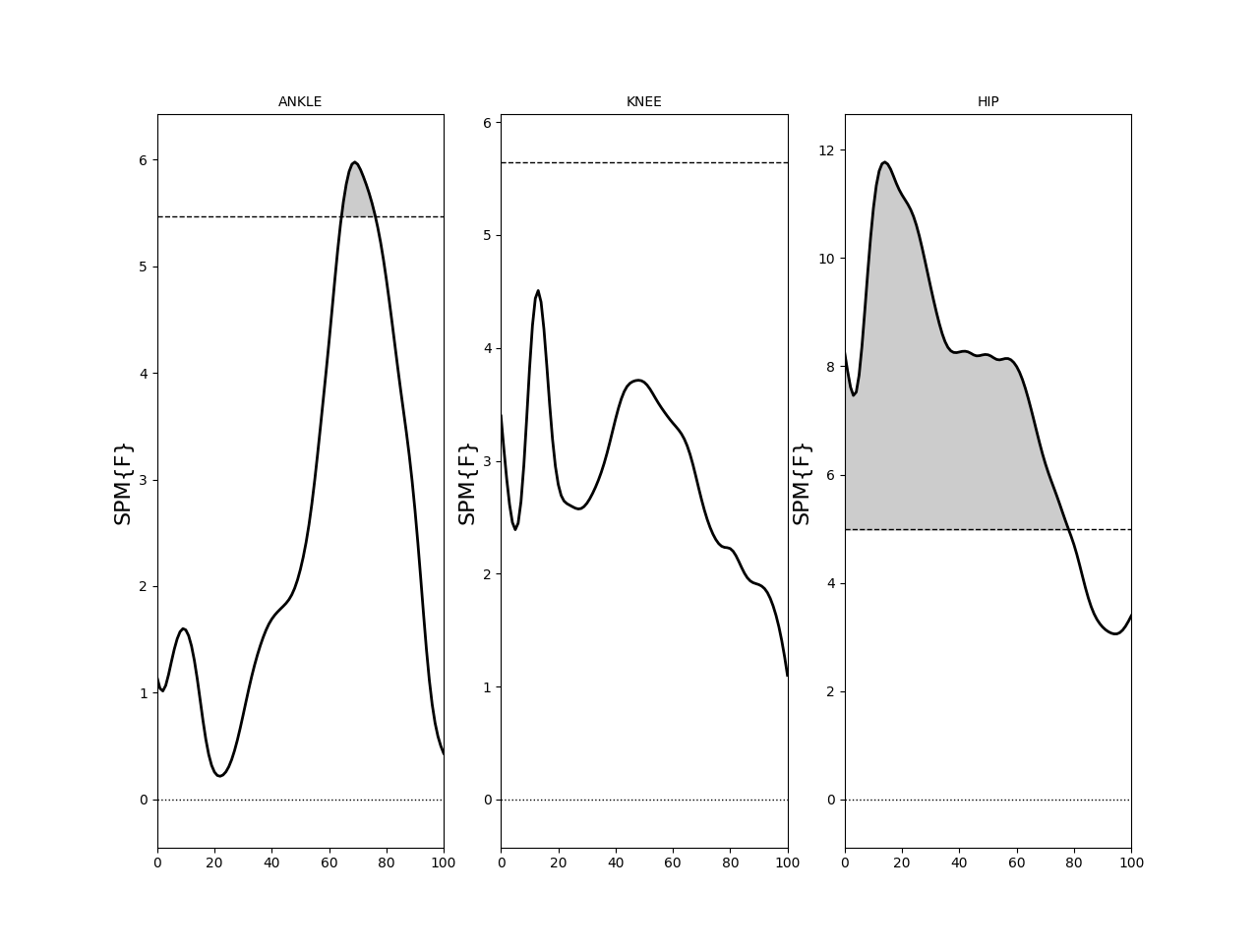


Supplementary figure 15: SPM ANOVA for the stair descent joint moment. Dashed line equivalent to α=0.05.


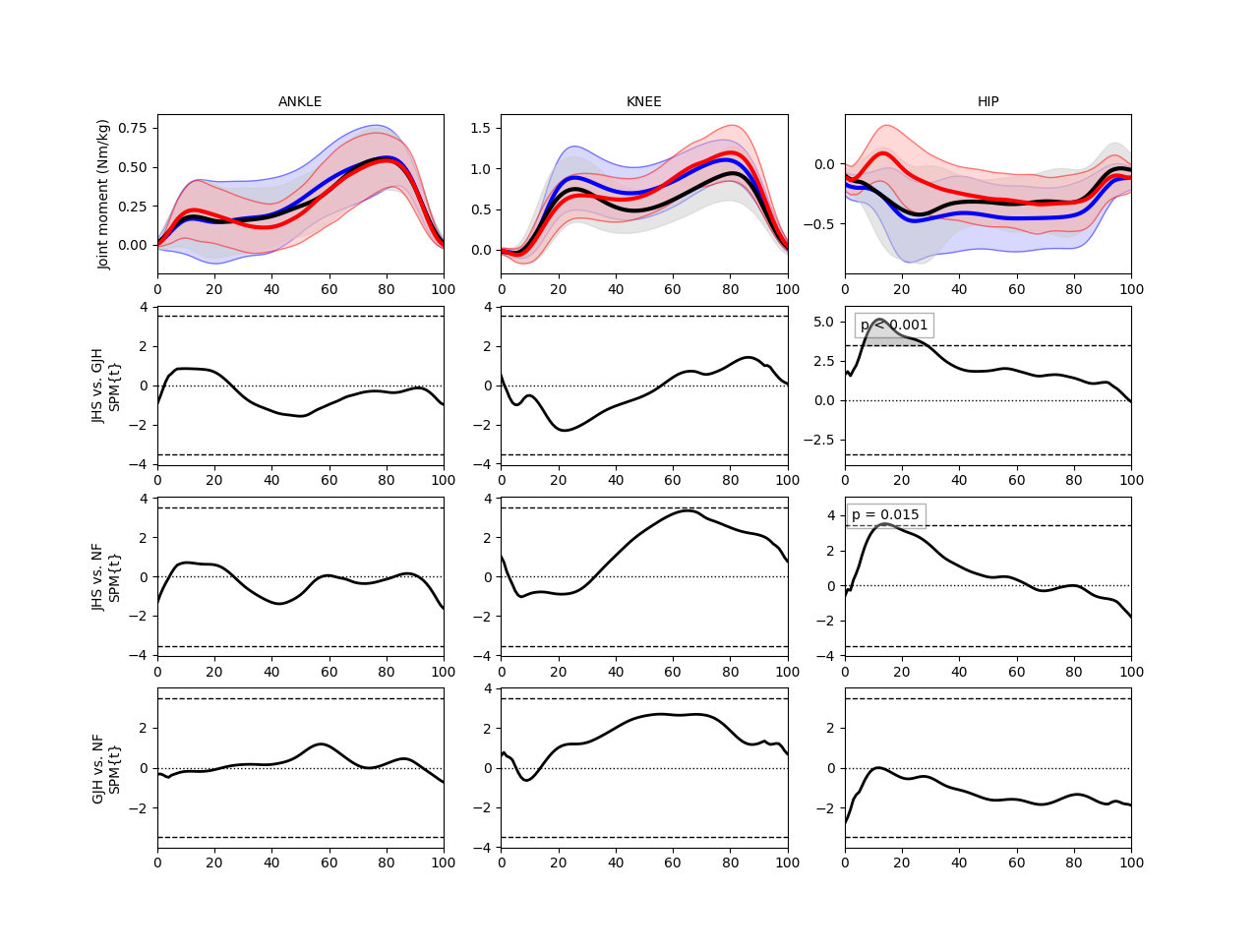


Supplementary figure 16. SPM post-hoc t-tests for stair descent joint moment. Horizontal axis is percent stance phase. Top row is mean joint moment ± 1 standard deviation for people with JHS (red), people with GJH (blue), and NF (black). Second to fourth rows show SPM{T} value throughout stance phase. Dashed lines equivalent to α=0.0169.


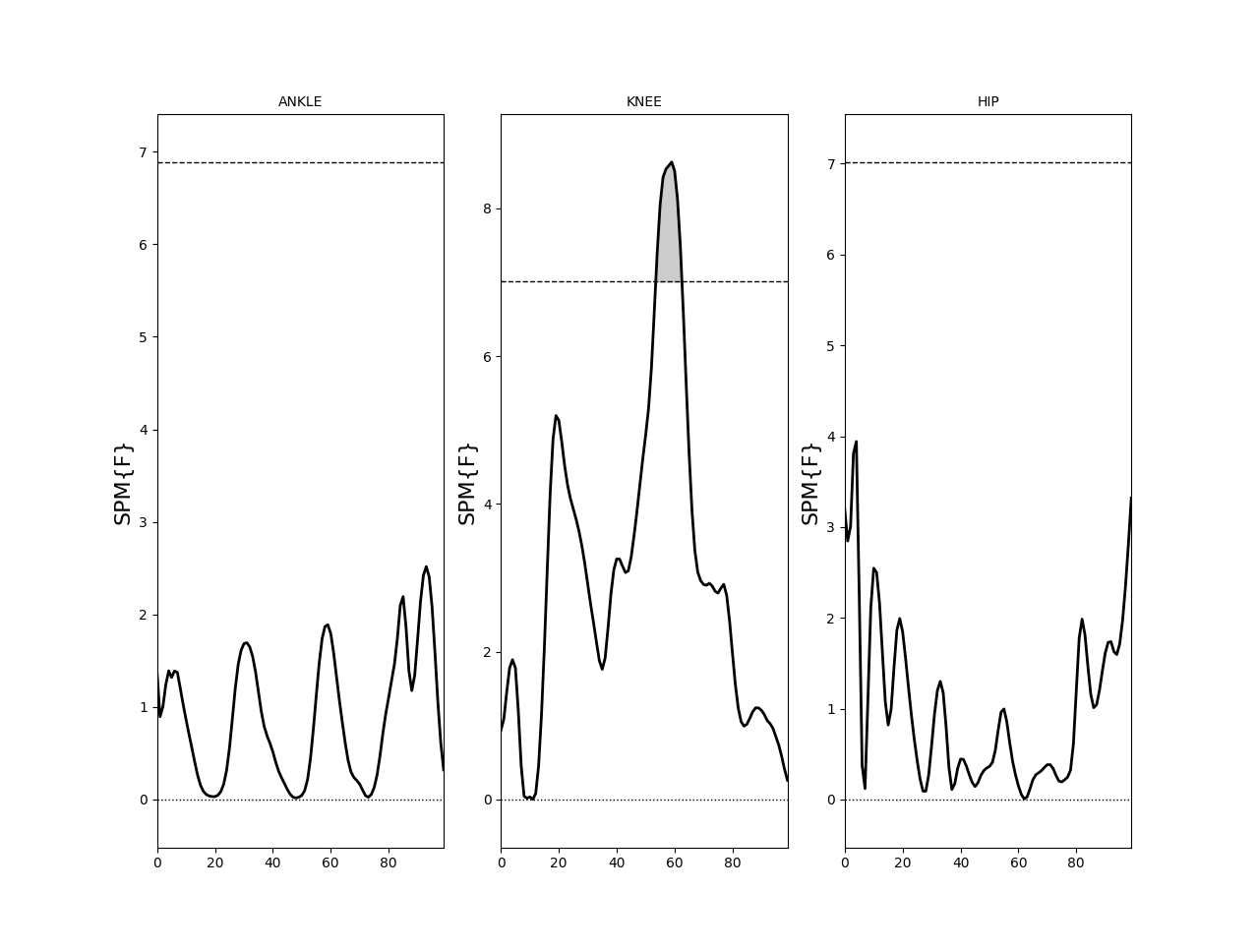


Supplementary figure 17: SPM ANOVA for the stair descent joint power. Dashed line equivalent to α=0.05.


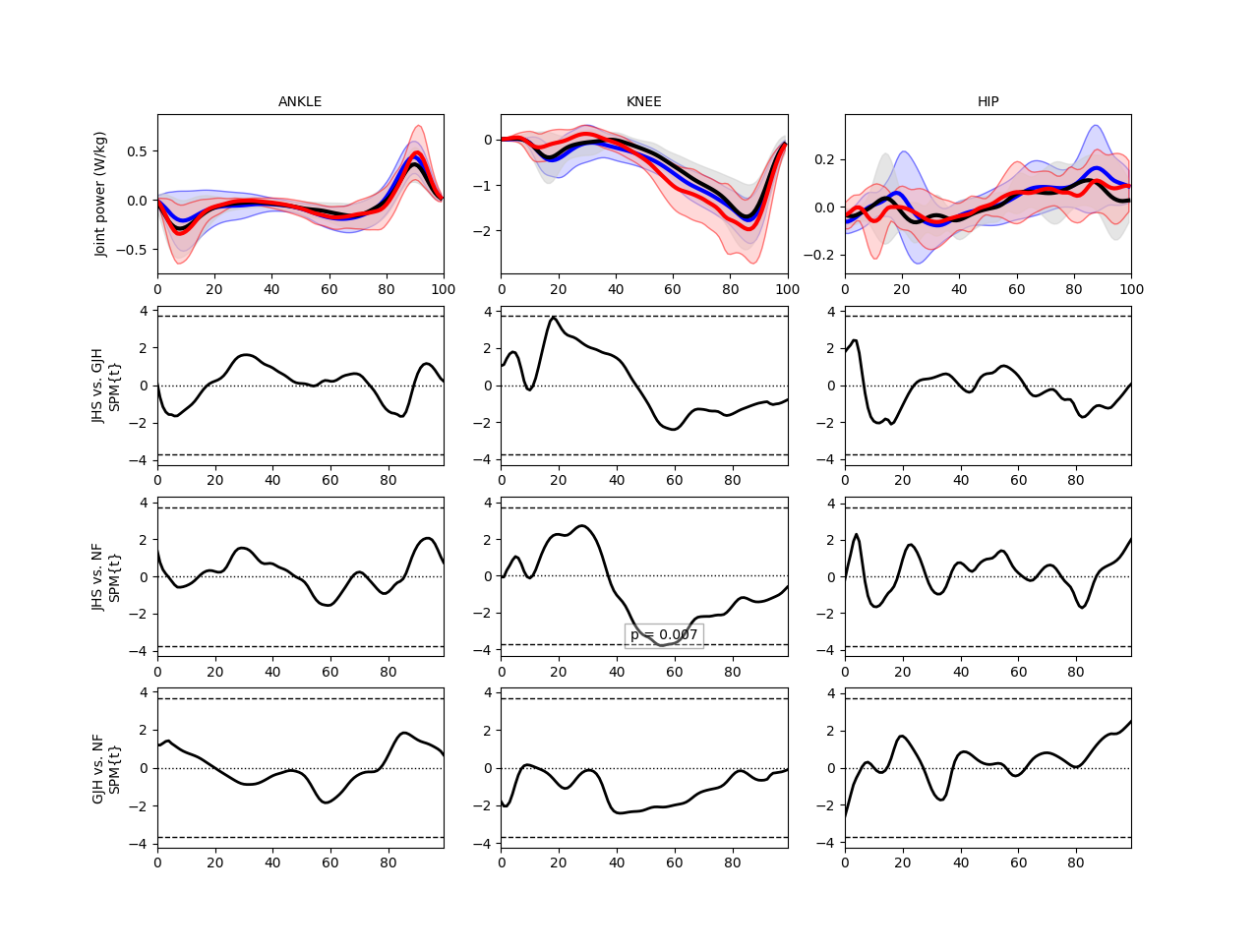


Supplementary figure 18. SPM post-hoc t-tests for stair descent joint power. Horizontal axis is percent stance phase. Top row is mean joint power ± 1 standard deviation for people with JHS (red), people with GJH (blue), and NF (black). Second to fourth rows show SPM{T} value throughout stance phase. Dashed lines equivalent to α=0.0169.
